# Geopositioning time series from offshore platforms in the Adriatic Sea

**DOI:** 10.1038/s41597-020-00705-w

**Published:** 2020-11-04

**Authors:** Mimmo Palano, Giuseppe Pezzo, Enrico Serpelloni, Roberto Devoti, Nicola D’Agostino, Stefano Gandolfi, Federica Sparacino, Letizia Anderlini, Luca Poluzzi, Luca Tavasci, Paolo Macini, Grazia Pietrantonio, Federica Riguzzi, Ilaria Antoncecchi, Francesco Ciccone, Giada Rossi, Antonio Avallone, Giulio Selvaggi

**Affiliations:** 1 Istituto Nazionale di Geofisica e Vulcanologia, Osservatorio Etneo - Sezione di Catania, P.zza Roma 2, I-95125 Catania, Italy; 2 Istituto Nazionale di Geofisica e Vulcanologia, Osservatorio Nazionale Terremoti, Via di Vigna Murata 605, I-00143 Rome, Italy; 3 University of Bologna, Department of Civil, Chemical, Environmental and Materials, Engineering (DICAM), Viale Risorgimento 2, Bologna, I-40136 Italy; 4 Istituto Nazionale di Geofisica e Vulcanologia, Sezione di Bologna, Via Donato Creti, 12, Bologna, I-40128 Italy; 5 Ministero dello Sviluppo Economico - DGISSEG, Via Molise 2, I-00187 Rome, Italy; 6 Research on Energy System RSE S.p.A., Via Rubattino 54, I-20134 Milano, Italy; 7 Istituto di Scienze Marine, Consiglio Nazionale delle Ricerche, Via P. Gobetti 101, I-40129 Bologna, Italy

**Keywords:** Energy security, Natural hazards, Geophysics, Geodynamics

## Abstract

We provide a dataset of 3D coordinate time series of 37 continuous GNSS stations installed for stability monitoring purposes on onshore and offshore industrial settlements along a NW-SE-oriented and ~100-km-wide belt encompassing the eastern Italian coast and the Adriatic Sea. The dataset results from the analysis performed by using different geodetic software (Bernese, GAMIT/GLOBK and GIPSY) and consists of six raw position time series solutions, referred to IGb08 and IGS14 reference frames. Time series analyses and comparisons evidence that the different solutions are consistent between them, despite the use of different software, models, strategy processing and frame realizations. We observe that the offshore stations are subject to significant seasonal oscillations probably due to seasonal environmental loads, seasonal temperature-induced platform deformation and hydrostatic pressure variations. Many stations are characterized by non-linear time series, suggesting a complex interplay between regional (long-term tectonic stress) and local sources of deformation (e.g. reservoirs depletion, sediment compaction). Computed raw time series, logs files, phasor diagrams and time series comparison plots are distributed via PANGAEA (https://www.pangaea.de).

## Abstract (summary)

**Table:** 

Measurement(s)	ground deformation process
Technology Type(s)	GPS navigation system
Factor Type(s)	GNSS station • temporal interval
Sample Characteristic - Location	Adriatic Sea

Machine-accessible metadata file describing the reported data: 10.6084/m9.figshare.13034372

## Background & Summary

**Fig. 1:**
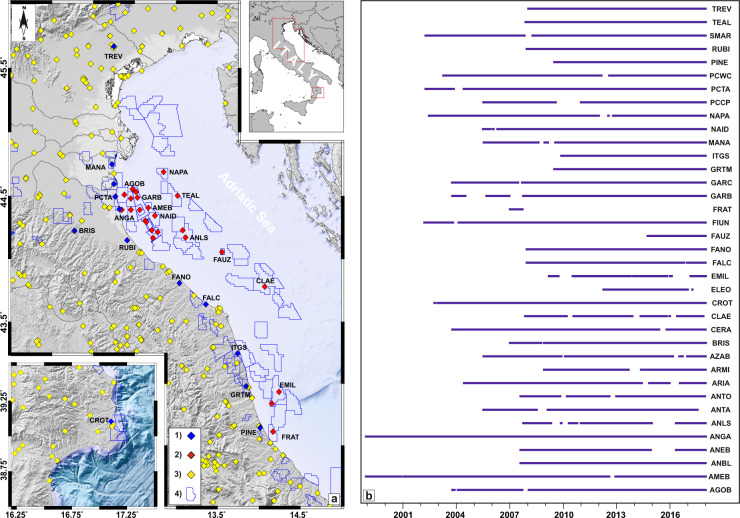
(**a**) Map distribution of the continuous GNSS stations provided by Eni S.p.A. Legend: 1) GNSS stations installed on storage and treatment centers, 2) GNSS stations installed on offshore platforms, 3) continuous GNSS stations developed and managed by different local, national and international Institutions and Agencies (INGV, ASI, ITALPOS, NETGEO, etc), 4) Oil/Gas prospection and production concession. (**b**) Temporal raw data availability of the GNSS dataset (see Table 1 for additional details).

We present the results from the analysis of a new dataset of GNSS stations installed on onshore and offshore industrial settlements provided by Eni S.p.A. (https://www.eni.com). This is one of the research activities started by INGV in the framework of the program “CLYPEA: Innovation network for future energy”, as part of the “subsoil deformations” project^1^. Such a dataset has been collected at continuous GNSS stations installed, respectively closely to 13 onshore infrastructures (e.g. storage centers) located along the Italian Adriatic coast and 24 offshore hydrocarbon production platforms anchored to the seabed in central and northern Adriatic Sea (Fig. 1a).

This dataset improves the current density of GNSS stations along the Adriatic coastal belt, allowing also to capture local and/or regional crustal deformation on a large sector of the northern Adriatic Sea, therefore representing a potentially invaluable dataset, although it involves GNSS stations not realized for geophysical purposes. Moreover, due to its offshore extension, the dataset must be considered as unique since until now, with the exclusion of very few GNSS stations (such as the HARV station installed on the Harvest platform, approximately located 10 km off the coast of central California^2^), not so extensive offshore datasets have been publicly released.

The raw GNSS dataset covers a period ranging from 1998 to the end of 2017, with time series covering time spans from 0.71 to 19.08 years (Table 1 and Fig. 1b). Raw data were provided by Eni S.p.A. in the RINEX format with 30-second sampling rate and containing 24 hours (00:00–23:59 UTC) of continuous tracking of NAVSTAR GPS (NAVigation Satellite Timing And Ranging Global Positioning System) satellites. In addition, Eni S.p.A. provided also a few ancillary information related to adopted receiver and antenna models and their changes over the acquisition time.

**Table 1:** GNSS dataset provided by Eni S.p.A. For each station, site id, denomination, notes (P, Platform; STC; Storage and treatment center), coordinates, installation date and duration are reported.

Site	Name	Note	Long. (°)	Lat. (°)	Heigth (meters)	Starting	Duration (years)
AGOB	AGOSTINO-B	P	12.472	44.554	67.13	2003.78	14.22
AMEB	AMELIA-B	P	12.662	44.407	73.26	1998.92	19.08
ANBL	ANNABELLA	P	13.079	44.229	76.63	2007.60	10.40
ANEB	ANEMONE-B	P	12.705	44.229	63.54	2007.60	10.40
ANGA	ANGELA-ANGELINA	P	12.344	44.391	73.34	1998.92	19.08
ANLS	ANNALISA	P	13.114	44.171	63.08	2007.76	10.24
ANTA	ANTARES	P	12.454	44.390	73.11	2005.52	12.03
ANTO	ANTONELLA	P	12.777	44.214	68.06	2007.60	10.37
ARIA	ARIANNA-A	P	12.628	44.306	68.84	2004.42	13.58
ARMI	ARMIDA	P	12.453	44.480	73.03	2008.92	9.07
AZAB	AZALEA-B	P	12.720	44.167	78.15	2005.53	12.47
BRIS	BRISIGHELLA	STC	11.774	44.225	220.68	2007.00	11.00
CERA	CERVIA-A	P	12.639	44.294	67.14	2003.78	14.22
CLAE	CLARA-EST	P	14.072	43.780	65.87	2007.85	10.02
CROT	CROTONE	STC	17.106	39.105	45.78	2002.77	15.23
ELEO	ELEONORA	P	14.156	42.840	72.53	2012.26	4.99
EMIL	EMILIO	P	14.243	42.935	66.18	2009.19	8.81
FALC	FALCONARA	STC	13.358	43.640	46.38	2007.95	10.05
FANO	FANO	STC	13.041	43.809	51.39	2007.94	10.06
FAUZ	FAUZIA	P	13.554	44.056	67.00	2014.74	3.26
FIUN	FIUMI UNITI	STC	12.316	44.397	43.59	2002.20	15.80
FRAT	FRATELLO CLUSTER	P	14.168	42.612	63.69	2007.00	0.71
GARB	GARIBALDI-B	P	12.532	44.487	66.39	2003.77	14.23
GARC	GARIBALDI-C	P	12.515	44.531	72.60	2003.77	14.23
GRTM	GROTTAMMARE	STC	13.845	42.980	70.57	2009.50	8.50
ITGS	ITALGAS	STC	13.744	43.245	105.91	2009.92	8.08
MANA	MANARA	STC	12.226	44.750	44.19	2005.53	12.47
NAID	NAIDE	P	12.745	44.343	62.33	2005.49	12.51
NAPA	NAOMI-PANDORA	P	12.847	44.689	68.83	2002.46	15.53
PCCP	PORTO CORSINI M E C	P	12.561	44.391	71.99	2005.52	12.48
PCTA	SPINARONI	STC	12.267	44.495	45.16	2002.28	15.72
PCWC	PORTO CORSINI M W C	P	12.373	44.509	72.42	2003.27	14.73
PINE	PINETO	STC	14.014	42.644	55.97	2009.50	8.50
RUBI	RUBICONE	STC	12.409	44.150	47.30	2007.95	10.05
SMAR	SMARLACCA	STC	12.247	44.594	44.43	2002.28	15.72
TEAL	TEA-LAVANDA-ARNICA	P	13.019	44.501	66.16	2007.88	10.12
TREV	TREVISO	STC	12.246	45.668	69.71	2008.04	9.96

Here we provide 3D coordinate time series on a daily basis, as processed by different data analysis centers, providing a robust validation of this particular set of positions time series. Coordinates are consistent with IGb08^3^ and IGS14^4^ reference frames. Both reference frames have origin coinciding with the Earth system center of mass, however, the IGS14 and the IGb08 represent the GNSS realization of the ITRF2014^5^ and ITRF2008^6^ reference frames, respectively. The data files are distributed in text ASCII format (i.e. the “pos” format^7^, realized by the Plate Boundary Observatory for GNSS time series solutions sharing) via a web-based online repository.

## Methods

This section illustrates the analyses carried out by different data processing centers, which analyzed the same dataset by means of different geodetic software and, within the same software adopted different procedures for the reference frames realization. With the aim of producing the most accurate final computation and, at the same time, obtaining a comparable analysis strategy, each data analysis center processed the raw GNSS data by using (i) a double difference approach, applied using the Bernese software, (ii) a double-difference distributed approach, applied by means of the GAMIT/GLOBK software package and (iii) a Precise Point Positioning (PPP) approach, applied by means of GIPSY.

The GNSS raw dataset has been processed at four different analysis centers:

- Istituto Nazionale di Geofisica e Vulcanologia - Sezione di Bologna (hereinafter INGV-BO); this analysis center provided 1 solution processed with GAMIT/GLOBK and QOCA (named *IBO_GAMIT*).
- Istituto Nazionale di Geofisica e Vulcanologia - Osservatorio Etneo (hereinafter INGV-OE); this analysis center provided 1 solution processed with GAMIT/GLOBK (named *IOE_GAMIT*).
- Istituto Nazionale di Geofisica e Vulcanologia - Osservatorio Nazionale Terremoti (hereinafter INGV-ONT); this analysis center provided 1 solution processed with GIPSY (named *ONT_GIPSY*) and 1 solution processed with Bernese (named *ONT_BERNESE*).
- Dipartimento di Ingegneria Civile, Chimica, Ambientale e dei Materiali - Università di Bologna (hereinafter DICAM-UniBO); this analysis center provided 1 solution processed with GIPSY (named *UBO_GIPSY*) and 1 solution processed with GAMIT/GLOBK (named *UBO_GAMIT*).

In the following, a brief description of each adopted procedure for obtaining daily positions is provided. Table 2 resumes some relevant characteristics of each processing scheme, including specific models applied in the analysis.

**Table 2:** Data processing parameters adopted during the processing of the GNSS dataset provided by Eni S.p.A.

Solution	UBO_GAMIT	UBO_GIPSY	IBO_GAMIT	IOE_GAMIT	ONT_GIPSY	ONT_BERNESE
**Data processing parameters**						
**Software**	**Gamit** (Ver. 10.61)	**GipsyX** (Ver. rc0.4)	**Gamit** (Ver. 10.70) + QOCA	**Gamit/Globk** (Ver. 10.70)	**Gipsy** (ver. 6.3)	**Bernese** (ver 5.0)
**Processing sampling rate**	30 sec	300 sec	30 sec	30 sec	30 sec	30 sec
**Elevation mask**	10°	10°	10°	10°	0°	10°
**Antenna phase center models**	igs08.atx	igs14_wwww.atx	igs08.atx	igs14_wwww.atx	igs14_wwww.atx	igs08.atx
**GPS orbits**	IGS, final	JPL, final	IGS, final	IGS, final	JPL, final	repro2, IGS, final
**Ocean tides loading model**	FES2004	FES2004	FES2004	FES2004	FES2004	FES2004
**Tropospheric mapping function**	VMF1	VMF1	VMF1	VMF1	VMF1	NIELL
**Ionosphere correction**	2nd order ionosphere correction	2nd order ionosphere correction	2nd order ionosphere correction	2nd order ionosphere correction	2nd order ionosphere correction	no 2nd order correction
**Ambiguity resolution**	Yes	Yes, with wlpb files	Yes	Yes	Yes, with wlpb files	Yes
**Reference frame/system and strategy for alignment of solutions**						
**Alignment strategy**	7 parameter transformation	7 parameter transformation	7 parameter transformation	7 parameter transformation	7 parameter transformation	4 parameter transformation
**Reference frame/system**	IGb08	IGb08	IGb08	IGb08	IGS14	IGb08
**Sinex file**	IGb08.snx	IGb08.snx	IGb08.snx	IGb08.snx		IGb08.snx
**Reference stations**	BUCU, GRAS, GRAZ, MATE, MEDI, NOT1, SOFI, WTZR, ZIMM	BUCU, GRAS, GRAZ, MATE, MEDI, NOT1, SOFI, WTZR, ZIMM	IGb08 core sites	BRUS, BRUX, COMO, GENO, GRAS,GRAZ, JOZE, MATE, NOT1, PRAT, TORI, ZIMM, ZOUF	132 stations used for reference frame alignment	45 anchor stations in Europe

### Raw data processing by using the Bernese software

The Bernese^8^ GNSS software is a scientific, high-precision, multi-GNSS data processing software developed at the Astronomical Institute of the University of Bern. Data processing with version 5.0 of this software package was performed at INGV-ONT. Daily solutions of station positions were estimated in loosely constraint reference systems. The raw observations were processed forming Ionosphere Free linear combinations and solving for the troposphere biases and phase ambiguities using the Quasi Ionosphere-free approach^9^. The troposphere modeling consisted in an a priori dry-Niell model fulfilled by the estimation of zenith delay corrections at 1-hour intervals at each site using the wet-Niell mapping function (see Table 2). In addition, one horizontal gradient parameter per day at each site was estimated. Ocean loading was computed using the FES2004 tidal model coefficients as provided by the Ocean Tide Loading provider (http://holt.oso.chalmers.se/loading). The GPS orbits and the Earth’s orientation parameters were fixed to the final IGS products and the site coordinates were constrained to an apriori sigma of 10 m, thus the daily coordinates were estimated in a loosely constrained, unknown reference frame. In order to express the *ONT_BERNESE* solution in a unique reference frame, the daily covariance was first projected imposing tight internal constraints (at millimeter level), and then the coordinates were transformed into the ITRF2008 reference frame by a 4-parameter Helmert transformation (translations and scale factor). The regional reference frame transformation used 45 anchor sites among the IGb08 stations, located on the Eurasian Plate (see Table 2).

### Raw data processing by using the GAMIT/GLOBK software

GAMIT/GLOBK^10^ is a GNSS analysis package, designed to run under any UNIX operating system and developed at the Massachusetts Institute of Technology (MIT), the Harvard-Smithsonian Center for Astrophysics, Scripps Institution of Oceanography and Australian National University. This software was adopted at DICAM-UniBO (version 10.61) and at INGV-BO and INGV-CT (version 10.7). All the three analysis centers adopted the IGS “Repro2 campaign”^11^ standards during the raw dataset processing. In this step, INGV-CT and DICAM-UniBO included into the cluster processing some high-quality IGS^12^ stations (Table 2) in order to improve the overall configuration of the network and to tie the regional stations to an external global reference frame. The INGV-BO solution is part of a continental-scale geodetic analysis, including >3000 continuous GNSS stations^13,14^. Because of this large number of sites, the GAMIT analysis was performed independently for several sub-networks, each made by <50 stations, with each sub-network sharing a set of high-quality IGS stations, which are used as tie-stations in the combination step.

During the processing step, the GAMIT software uses an ionosphere-free linear combination of GNSS phase observables by applying a double differencing technique to eliminate phase biases related to drifts in the satellite and receiver clock oscillators. GPS phase data were weighted according to an elevation-angle-dependent error model^10^ using an iterative analysis procedure whereby the elevation dependence was determined by the observed scatter of phase residuals. In this analysis the parameters of satellites orbit were fixed to the IGS final products. IGS absolute antenna phase center models (igs08.atx and igs14_wwww.atx available at ftp://ftp.igs.org/pub/station/general/; see also Table 2) for both satellite and ground-based antennas were adopted in order to improve the accuracy of vertical site position component estimations^15,16^. The first-order ionospheric delay was eliminated by using the ionosphere-free linear combination, while a second-order ionospheric corrections^17^ were applied using the IONEX files from the Center for Orbit Determination in Europe (CODE). The tropospheric delay was modeled as a piecewise linear model and estimated using the Vienna Mapping Function 1 (VMF1^18^) with a 10° cutoff. The Earth Orientation Parameters (EOP) were tightly constrained to priori values obtained from IERS Bulletin B. The ocean tidal loading was corrected using the FES2004^19^ model. The International Earth Rotation Service (IERS) 2003 model for diurnal and semidiurnal solid Earth tides was also adopted.

In a successive step, the GAMIT solutions, in the form of loosely-constrained H-files, were aligned to the IGb08 reference frame. Such an alignment was performed by INGV-CT and DICAM-UniBO through the GLOBK/GLORG^10^ software package by minimizing the deviations between horizontal positions and velocities of achieved solutions and those available in the IGb08.snx^20^ reference solutions (Table 2). INGV-BO, instead, performed the alignment to the IGb08 reference frame through the ST_FILTER program of the QOCA^21,22^ software and by combining all the daily loosely constrained solutions with the global solution of the IGS network made available by MIT (ftp://everest.mit.edu/pub/MIT_GLL/), and simultaneously realizing a global reference frame by applying generalized constraints^21^. Specifically, the reference frame was defined by minimizing the velocities of the IGS core^23^ stations, while estimating a seven-parameter transformation with respect to the GNSS realization of the ITRF2008^6^ frame, i.e., the IGb08^3^ reference frame.

### Raw data processing by using the GIPSY software

GIPSY is a GNSS-Inferred Positioning System and Orbit Analysis Simulation software package (https://gipsy-oasis.jpl.nasa.gov/) developed by the Jet Propulsion Laboratory. Data processing with this software package was performed at DICAM-UniBO (which used GIPSYX, version rc0.4) and INGV-ONT (which used GIPSY-OASIS II, version 6.3).

Raw observations were processed by both analysis centers in a precise point positioning mode applied to ionospheric-free carrier phase and pseudorange data^24^ and using JPL’s final fiducial-free GNSS orbit products. Ocean loading tidal loading and companion tides, computed using the FES2004^19^ tidal model coefficients (http://holt.oso.chalmers.se/loading) and were applied as a station motion model. The wet zenith troposphere and two gradient parameters were estimated every 5 minutes as a random walk process^25^ by using the VMF1^18^ with a 10° cutoff. A second-order ionospheric correction^26^ was applied during the data processing. Station clock errors was treated as a white-noise process. The ambiguity resolution was performed by using the wide lane and phase bias (WLPB) method^24^, which phase-connects individual stations to IGS stations in common view. Resolving ambiguities reduced significantly the scatter mostly in the east component of time series. Satellite orbits and clock parameters were provided by JPL who determine them in a global fiducial free analysis using a subset of the available IGS core stations as tracking sites.

In this step, the *ONT_GIPSY* solution was aligned to IGS14^4^ by applying a daily seven-parameter Helmert transformation (three rotations, three translations, and a scale component obtained from JPL) specifically calculated using the IGS14 Cartesian coordinates and velocities of 132 stations selected by specific quality criteria (e.g. long observation time, continuity through time, position and velocity constrained with sub-millimeter level accuracy, etc.) and located in and around the Eurasian plate and in the Mediterranean area^27^. This reference frame realization method applies a continental-scale spatial filter to the station coordinates, leading to a reduction of the common-mode errors^28^ and to an increase of the signal-to-noise ratio.

The *UBO_GIPSY* solution was aligned to the IGb08^3^ reference frame by applying a daily seven-parameter Helmert transformation which was ad hoc calculated using as reference a regional subset of IGS tracking network (see Table 2). The reference coordinates of this regional subset can be found in the IGb08.snx^20^ file provided by IGS.

Each analysis center, in the end, provided position time-series in a common format (e.g. the UNAVCO PBO “pos” ASCII file format^7^); the *ONT_GIPSY* solution was aligned to IGS14 while the other solutions were aligned to IGb08. It must be noted that some few stations show time series with different temporal length because each analysis center requested the RINEX dataset to Eni S.p.A. at different times. Moreover, the *ONT_GIPSY* solution spans the 2000.0–2017.99 time interval since the limited number of continuous stations doesn’t allow to adequately constraint the adopted continental-scale spatial filter prior to 2000.

## Data Records

All the computed raw time series are distributed via the PANGEA repository^29^ so that the interested researchers can use them for future studies. The raw time series (in ASCII pos format) were stored in different folders and accordingly renamed, based on the solutions described in paragraph “Raw data processing”.

We included in the same repository:^29^

- A file, named “ENI_Offsets.json”, containing all manually picked offsets; as mentioned above it can be used as input for later use in the TSAnalyzer software^24^.
- All logs files as resulting from the analysis performed with the TSAnalyzer software; the logs files were renamed accordingly to the associated solution. These logs contain all the parameters discussed in section 3.
- The phasor diagrams in png format.
- The time series plots (observed, modelled and residual components) in png format; the plots were renamed accordingly to the associated solution.
- The time series comparison plots in png format.

The original raw RINEX data are property of Eni S.p.A. company. Interested users can obtain free access to data under a specific confidential agreement by contacting Eni S.p.A. company (see the Supplementary Information for details).

## Technical Validation

As mentioned above, the position time series computed by each analysis center as well as the estimated velocity fields were compared in order to highlight possible differences arising from the use of the different software and from the specific models that were selected and applied in the analysis.

### Position time series comparison

To perform a simple comparison between the position time series computed by each analysis center we used the TSAnalyzer^30,31^ open-source software which reads GNSS position time series with different formats and allows to remove outliers as well as to simultaneously estimate linear and seasonal signals.

Roughly speaking, position time series can be modelled as the superposition of three main types of signals: (i) linear long-term deformation, (ii) seasonal signals, (iii) instrumental (e.g., GNSS antenna changes) and tectonic (co-seismic displacement) offsets. Therefore, site motion *y(t)* for each component can be modelled as:

1 $$y({t}_{i})=a+b{t}_{i}+c\,{\sin }(2\pi {t}_{i})+d\,{\cos }(2\pi {t}_{i})+e\,{\sin }(4\pi {t}_{i})+f\,{\cos }(4\pi {t}_{i})+\mathop{\sum }\limits_{j=1}^{{n}_{g}}\,{g}_{i}H({t}_{i}-{T}_{gj})+{v}_{i}$$

where t_i_ for *i* = *1…N* are the daily solution epochs (in units of years). The terms *a* and *b* are the site position and the linear rate; coefficients *c* and *d* describe the annual periodic motion while *e* and *f* stand for the semi-annual motion. The summation term corrects for any number (n_g_) of offsets, with magnitudes *g* and epoch *T*_*g*_ using the Heaviside^32^ step function H and the last term, ν_i_, is the residual.

A first visual inspection of all raw coordinates time series reveals that they contain both offsets and outliers. Outliers showing large uncertainties (e.g. blunders) were removed by setting in TSAnalyzer an arbitrary sigma criterion (we set 15, 15 and 20 mm, for North, East and Up components, respectively). Offsets were manually picked and recorded in the “ENI_Offsets.json” file (available on the repository) for later use (see also Table 3). Offsets classified as “tectonic” correspond to co-seismic deformation related to the M >6 earthquakes striking central Italy on 24 August 2016 and on 26 October 2016^33^. Offsets classified as “equipment” correspond to change in antennas while offsets classified as “unknown” are characterized by suspicious motion whose nature should be related to service operations on platform.

**Table 3:** Offsets manually picked and recorded in the “ENI_Offsets.json” file which is available on the repository.

Site	Offset date	Notes
AMEB	26/10/2011	unknown
	15/03/2012	unknown
	20/03/2012	unknown
	01/04/2012	unknown
	07/04/2012	unknown
	11/04/2012	unknown
	12/05/2012	unknown
	17/05/2012	unknown
	04/12/2012	equipment
ELEO	30/10/2016	tectonic
EMIL	29/07/2015	unknown
	25/01/2016	unknown
FALC	30/10/2016	tectonic
GARC	06/10/2011	unknown
GRTM	22/06/2010	unknown
	24/08/2016	tectonic
	30/10/2016	tectonic
	14/12/2017	unknown
ITGS	24/08/2016	tectonic
	30/10/2016	tectonic
NAPA	01/01/2012	unknown
PCWC	07/10/2009	unknown
	14/07/2011	unknown
PINE	24/08/2016	tectonic
	30/10/2016	tectonic
SMAR	31/08/2011	unknown

Finally, estimated parameters for each solution are reported as ASCII log files in the online repository. The following analysis is based on the results achieved with this time-series analysis.

**Fig. 2:**
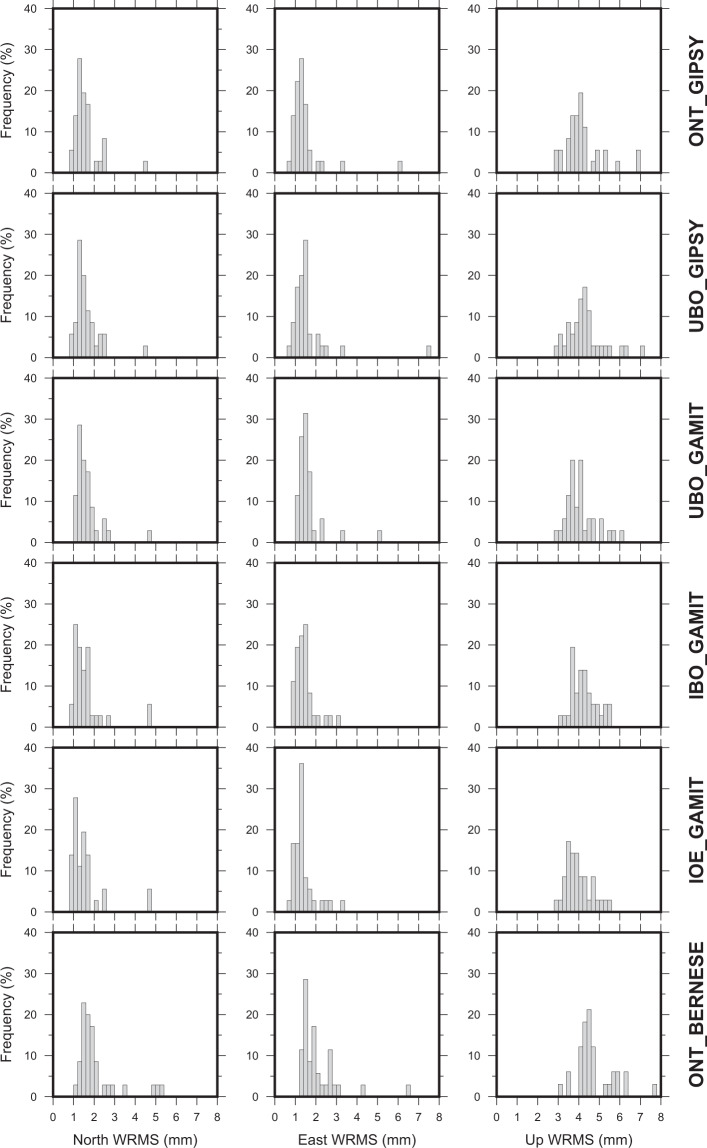
Frequency histograms of WRMS values for the North, East and Up components of filtered residual time series.

The Weighted Root Mean Squares (WRMS) values of all time-series were also estimated by using the TSAnalyzer software (see log files on the repository), after filtering outliers and estimating parameters according to Eq. (1). In such a step, each single daily solution was weighted with respect to its formal sigma without taking into account the correlation between the North, East and Up components. Frequency histograms of WRMS values for horizontal and vertical components of each different solution analyzed in this study are reported in Fig. 2, while range, mean and median values are reported in Table 4. All solutions are characterized by very similar values, highlighting similar patterns between the solutions as for instance, (i) the mean and median WRMS values for vertical component are about three times larger than the horizontal ones and (ii) the higher WRMS values are observed always at the same stations (CERA and AMEB; see the related logs file in the repository for additional details). Moreover, computed WRMS values are in the same range of the ones estimated for on-shore stations with comparable observing time windows^13,34^.

**Table 4:** Range, mean and median values (in mm) for time series residuals WRMS.

Solution	North			East			Up		
	*Range*	*Mean*	*Median*	*Range*	*Mean*	*Median*	*Range*	*Mean*	*Median*
IBO_GAMIT	0.94–4.79	1.64	1.40	0.93–3.17	1.49	1.39	3.06–8.01	4.37	4.20
IOE_GAMIT	0.88–4.96	1.55	1.28	0.75–3.26	1.39	1.28	2.85–8.67	4.12	3.87
ONT_BERNESE	1.02–5.21	2.09	1.73	1.21–6.43	2.08	1.84	3.18–9.40	5.00	4.55
ONT_GIPSY	0.89–4.43	1.58	1.42	0.70–6.05	1.48	1.27	2.95–6.95	4.21	4.05
UBO_GAMIT	1.08–4.63	1.66	1.44	1.04–5.18	1.65	1.49	2.91–6.08	4.08	3.97
UBO_GIPSY	0.94–4.54	1.64	1.49	0.80–7.49	1.63	1.41	2.96–7.06	4.33	4.27

**Fig. 3:**
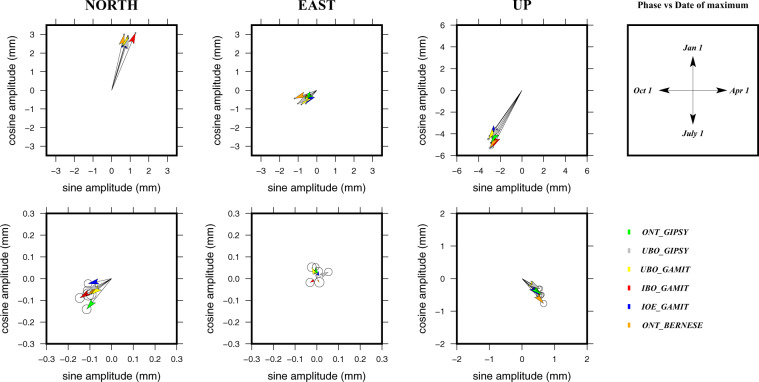
Example of phasor diagrams of annual (top) and semiannual (bottom) signals for station AGOB (see Table 1). Amplitudes of the estimated sine and cosine parameters (see Eq. (1)) are plotted for the North, East and Up components. The upper-right plot represents the key to correlate the maxima phase direction with the day year period. Phases are referred to January 1 and time increases clockwise.

All the examined time series contain significant seasonal signals in both horizontal and vertical directions. Values estimated for each solution show a large agreement between them; annual amplitudes range between 0.5–5.3 mm horizontally and 3–8 mm vertically with higher values observed at sites installed on the offshore platforms (see plots reported in the online repository). Semi-annual amplitudes are usually <0.5 mm horizontally and ~1 mm vertically. Additional features on seasonal signals are provided by considering phasor diagrams of amplitudes and phase signals (see the example reported in Fig. 3). Regarding the annual signals, most of the sites installed on the offshore platforms show maximum amplitudes on January, September and August for North, East and Up components, respectively. Moreover, most of these sites (especially those closely located to the coastal area) are subjected to horizontal oscillations with a prevailing NNW-SSE attitude and to vertical oscillation associated with uplift during summer and subsidence during winter. Sites installed along the coastal area are characterized by maximum amplitudes largely scattered and no clear oscillation patterns can be recognized. Concerning the semiannual signals, most of the sites show maximum amplitudes concentrated during July-September for North component and during April-June for both East and Up components.

Some position time series (mainly recorded at the stations located on the offshore platforms) exhibit a curved shape, while others show weak post-seismic decay (recorded at few stations located along the coastal area, eastward of the 2016 seismic sequence epicentral area). Both features result in deviations from the linear trend; to quantify such a derivation for each time series, we defined a simple non-linearity index (*I*_*NL*_) as the ratio between the standard deviation of the smoothed time series and the standard deviation of its intrinsic noise:

2 $${I}_{NL}=\frac{\sqrt{{\sum }_{i=1}^{N}\left|{F}_{i}-\bar{F}\right|}}{\sqrt{{\sum }_{i=1}^{N}\left|{R}_{i}-\bar{R}\right|}}$$

where *i* = *1…N* is the number of data, F and R (with mean values $\bar{F}$ and $\bar{R}$) are the smoothed and the residual observations. The smoothed time series should approximate the low frequency non-linear behavior, while the residual time series (computed as the difference between the unsmoothed and the smoothed time series) would contain most of the high frequency noise and should be regarded as a good indicator of the station intrinsic noise. Therefore, by taking into account the time series already de-trended with respect to Eq. (1), we computed i) the smoothed time series by applying a moving average filter with a time span of 1 year, ii) the related residual time series and finally, iii) the *I*_*NL*_ values (Table 5). Estimations of *I*_*NL*_ index on a set of high-quality IGS stations (Table 2) highlight that values <0.5 reflect time series with pure linear behavior, therefore in the following we arbitrarily set as “gentle” and “moderately” non-linear all the time series with 0.5 ≤ *I*_*NL*_ ≤ 1 and *I*_*NL*_ > 1, respectively (Table 5). Based on this simple definition and excluding FRAT station because of its short time series, 11 stations are generally characterized by linear time series (for all the three components) while the other are characterized by gentle (19 stations) and moderately (6 stations) non-linear time series, at one or more components. Stations with gentle and moderately non-linear time series mainly concentrates offshore on northern Adriatic Sea and onshore, along the coastal belt; the stations with linear time series are mainly located along the coastal onshore belt.

**Table 5:** Mean I_*NL*_ index values and associated standard deviation (based on the values computed for all the different time series solutions) for North, East and Up components.

Site	North	East	Up
AGOB	0.61 ± 0.08	0.41 ± 0.09	0.74 ± 0.07
AMEB	0.19 ± 0.08	0.55 ± 0.22	0.45 ± 0.18
ANBL	0.71 ± 0.11	0.61 ± 0.13	0.90 ± 0.12
ANEB	0.97 ± 0.25	0.34 ± 0.10	0.36 ± 0.14
ANGA	1.04 ± 0.39	1.09 ± 0.40	1.61 ± 0.69
ANLS	0.30 ± 0.05	0.51 ± 0.20	0.66 ± 0.21
ANTA	0.54 ± 0.06	0.76 ± 0.12	0.79 ± 0.17
ANTO	0.46 ± 0.06	0.39 ± 0.07	0.33 ± 0.08
ARIA	1.15 ± 0.13	0.35 ± 0.10	0.70 ± 0.10
ARMI	0.40 ± 0.05	0.53 ± 0.08	0.40 ± 0.05
AZAB	0.34 ± 0.07	0.33 ± 0.10	0.49 ± 0.06
BRIS	0.48 ± 0.19	0.39 ± 0.14	0.28 ± 0.04
CERA	2.48 ± 1.09	0.72 ± 0.47	0.50 ± 0.26
CLAE	1.82 ± 0.11	1.30 ± 0.28	0.37 ± 0.03
CROT	0.98 ± 0.23	0.47 ± 0.15	0.51 ± 0.05
ELEO	0.32 ± 0.08	0.24 ± 0.07	0.17 ± 0.05
EMIL	0.30 ± 0.09	0.34 ± 0.05	0.28 ± 0.04
FALC	0.36 ± 0.05	0.29 ± 0.03	0.26 ± 0.05
FANO	0.54 ± 0.03	0.48 ± 0.10	0.28 ± 0.10
FAUZ	0.10 ± 0.03	0.24 ± 0.05	0.32 ± 0.03
FIUN	1.44 ± 0.26	3.11 ± 1.11	1.26 ± 0.09
FRAT	0.02 ± 0.00	0.01 ± 0.00	0.01 ± 0.00
GARB	0.59 ± 0.14	0.71 ± 0.18	0.82 ± 0.07
GARC	0.58 ± 0.33	0.52 ± 0.42	0.67 ± 0.33
GRTM	0.21 ± 0.05	0.23 ± 0.03	0.17 ± 0.04
ITGS	0.77 ± 0.10	0.82 ± 0.10	0.33 ± 0.06
MANA	0.52 ± 0.09	0.55 ± 0.13	0.26 ± 0.04
NAID	0.40 ± 0.09	0.45 ± 0.06	0.53 ± 0.05
NAPA	0.40 ± 0.11	0.72 ± 0.14	0.29 ± 0.07
PCCP	0.42 ± 0.07	0.46 ± 0.13	0.40 ± 0.08
PCTA	1.16 ± 0.19	1.08 ± 0.30	0.79 ± 0.12
PCWC	0.36 ± 0.11	0.34 ± 0.07	0.77 ± 0.23
PINE	0.28 ± 0.04	0.23 ± 0.05	0.22 ± 0.04
RUBI	0.48 ± 0.05	0.54 ± 0.06	0.69 ± 0.07
SMAR	0.28 ± 0.07	0.45 ± 0.09	0.60 ± 0.08
TEAL	1.14 ± 0.09	1.10 ± 0.19	0.71 ± 0.13
TREV	0.26 ± 0.07	0.29 ± 0.07	0.23 ± 0.06

**Fig. 4:**
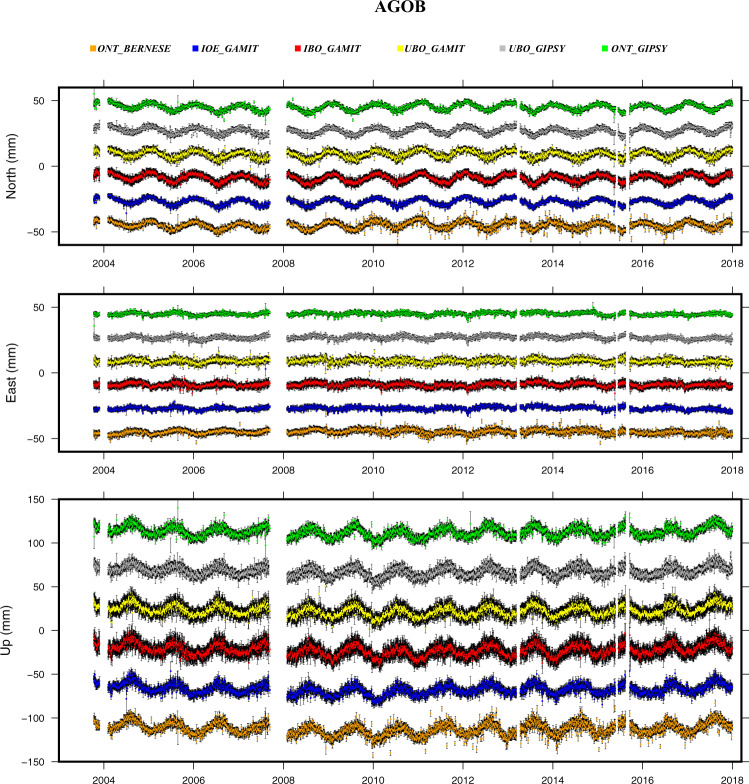
Example of time series comparison from the different solution described in the main text. Each time series is reported after correcting offsets and removing the linear trend as defined in Eq. (1).

As a last step, the time-series were compared between them after correcting the offsets (Table 3) and removing the linear trend as defined in Eq. (1). An example of such a comparison is reported in Fig. 4, while the remaining plots are reported in the online repository. This simple comparison highlights how the main features characterizing the time-series (e.g. seasonal signals, noisier time intervals, short-term transients, etc.) show a good agreement between the different solutions.

### Velocity field analysis and comparison

In the following, we performed some simple comparisons between all the computed linear trend values; however, these analyses (and related results) must be considered with caution because of the non-linear motion detected at many stations.

**Fig. 5:**
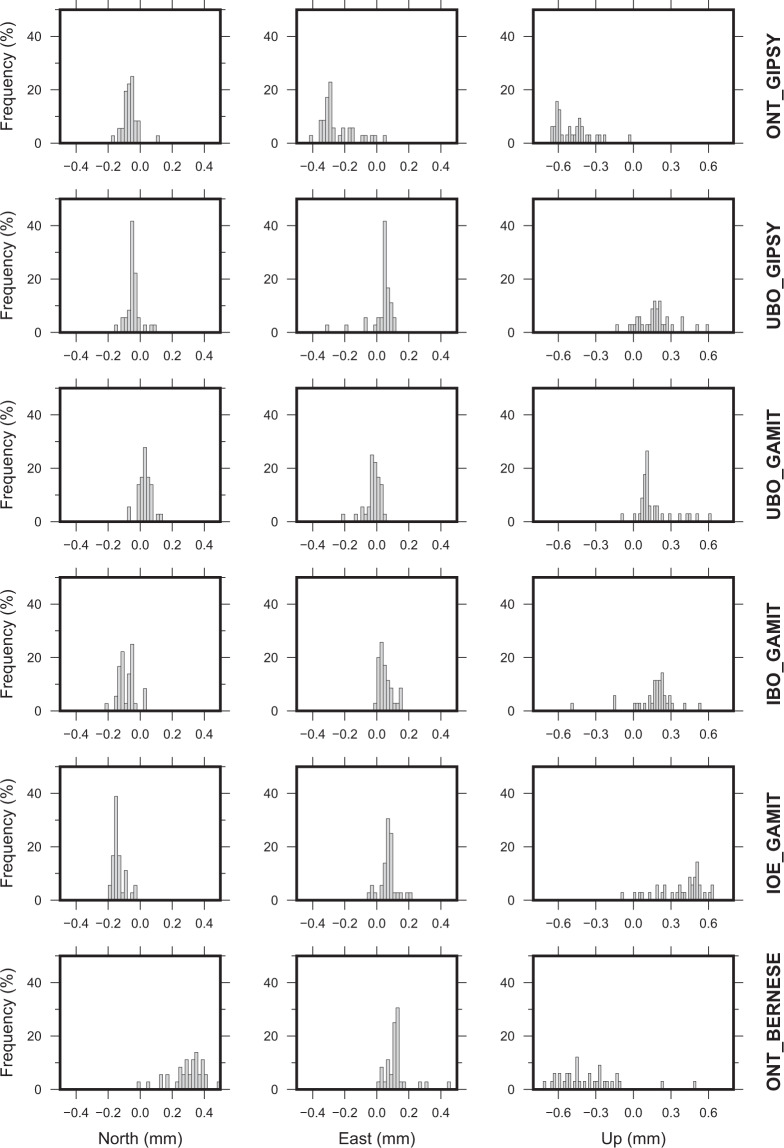
Frequency histograms of residual values between computed linear trend values with respect to the mean values (Table 5).

As a first step, we calculated the residual values with respect to the mean ones for each solution and for each component (Table 6). Results are reported as frequency histograms in Fig. 5. Regarding the North component, the IBO_GAMIT, the IOE_GAMIT and the ONT_GIPSY solutions show values up to 0.2 mm smaller than the mean values, while the ONT_BERNESE solution shows values up to 0.4 mm larger than the mean values; the UBO_GAMIT and UBO_GIPSY solutions show values centered (±0.1 mm) on the mean values. Regarding the East component, the IBO_GAMIT, the IOE_GAMIT and the ONT_BERNESE solutions show values up to 0.2 mm larger than the mean values, while the ONT_GIPSY solution shows values up to 0.4 mm smaller than the mean values; the UBO_GAMIT and UBO_GIPSY show again values centered (±0.1 mm) on the mean values. Regarding the Up component, the IBO_GAMIT, the IOE_GAMIT, the UBO_GAMIT and the UBO_GIPSY solutions show prevailing values up to 0.6 mm larger than the mean values, while the ONT_GIPSY and the ONT_BERNESE solutions show prevailing values up to 0.4 mm smaller than the mean values; the overall differences range in the −0.6 to 0.6 mm value interval.

**Table 6:** Mean linear rate values and associated standard deviation (in mm) for North, East and Up components. No estimates were performed for the FRAT site because of its short time series.

Site	North	East	Up
AGOB	16.77 ± 0.19	22.62 ± 0.12	−7.14 ± 0.42
AMEB	17.38 ± 0.24	20.52 ± 0.18	−3.07 ± 0.41
ANBL	18.32 ± 0.16	21.96 ± 0.15	−3.74 ± 0.38
ANEB	17.79 ± 0.06	20.98 ± 0.18	−4.80 ± 0.40
ANGA	19.41 ± 0.22	22.16 ± 0.14	−17.56 ± 0.52
ANLS	18.12 ± 0.17	21.18 ± 0.18	−4.23 ± 0.34
ANTA	18.14 ± 0.19	20.74 ± 0.16	−4.91 ± 0.35
ANTO	18.45 ± 0.16	21.34 ± 0.16	−2.26 ± 0.41
ARIA	17.84 ± 0.20	23.18 ± 0.18	−10.79 ± 0.56
ARMI	17.75 ± 0.13	21.94 ± 0.16	−4.39 ± 0.41
AZAB	18.65 ± 0.18	22.18 ± 0.15	−3.64 ± 0.39
BRIS	18.96 ± 0.17	23.09 ± 0.16	1.55 ± 0.39
CERA	18.32 ± 0.19	22.74 ± 0.13	−11.66 ± 0.36
CLAE	15.73 ± 0.15	21.25 ± 0.15	−14.31 ± 0.37
CROT	17.73 ± 0.18	27.24 ± 0.10	−0.24 ± 0.60
ELEO	17.98 ± 0.16	23.11 ± 0.10	0.08 ± 0.52
EMIL	18.23 ± 0.10	23.13 ± 0.21	−1.89 ± 0.31
FALC	18.46 ± 0.13	23.00 ± 0.17	−0.15 ± 0.38
FANO	18.91 ± 0.14	22.40 ± 0.16	0.15 ± 0.38
FAUZ	19.77 ± 0.20	20.55 ± 0.26	−5.65 ± 0.47
FIUN	15.56 ± 0.19	28.72 ± 0.09	−14.18 ± 0.43
GARB	17.77 ± 0.18	20.98 ± 0.14	−7.06 ± 0.42
GARC	17.15 ± 0.08	19.73 ± 0.08	−8.56 ± 0.28
GRTM	18.50 ± 0.10	23.68 ± 0.15	−0.30 ± 0.30
ITGS	19.07 ± 0.09	22.70 ± 0.14	0.13 ± 0.36
MANA	17.33 ± 0.17	21.59 ± 0.14	−2.98 ± 0.46
NAID	17.82 ± 0.17	20.51 ± 0.15	−3.53 ± 0.42
NAPA	17.56 ± 0.20	21.45 ± 0.08	−1.52 ± 0.34
PCCP	16.84 ± 0.18	23.29 ± 0.15	−4.14 ± 0.38
PCTA	19.65 ± 0.18	23.86 ± 0.11	−7.57 ± 0.40
PCWC	17.01 ± 0.21	22.11 ± 0.04	−5.30 ± 0.51
PINE	18.64 ± 0.10	23.18 ± 0.18	−0.74 ± 0.35
RUBI	19.39 ± 0.13	22.13 ± 0.16	−2.28 ± 0.36
SMAR	18.39 ± 0.21	20.94 ± 0.05	−6.54 ± 0.47
TEAL	18.21 ± 0.16	19.76 ± 0.16	−4.55 ± 0.39
TREV	17.23 ± 0.16	20.51 ± 0.15	−0.10 ± 0.44

**Fig. 6:**
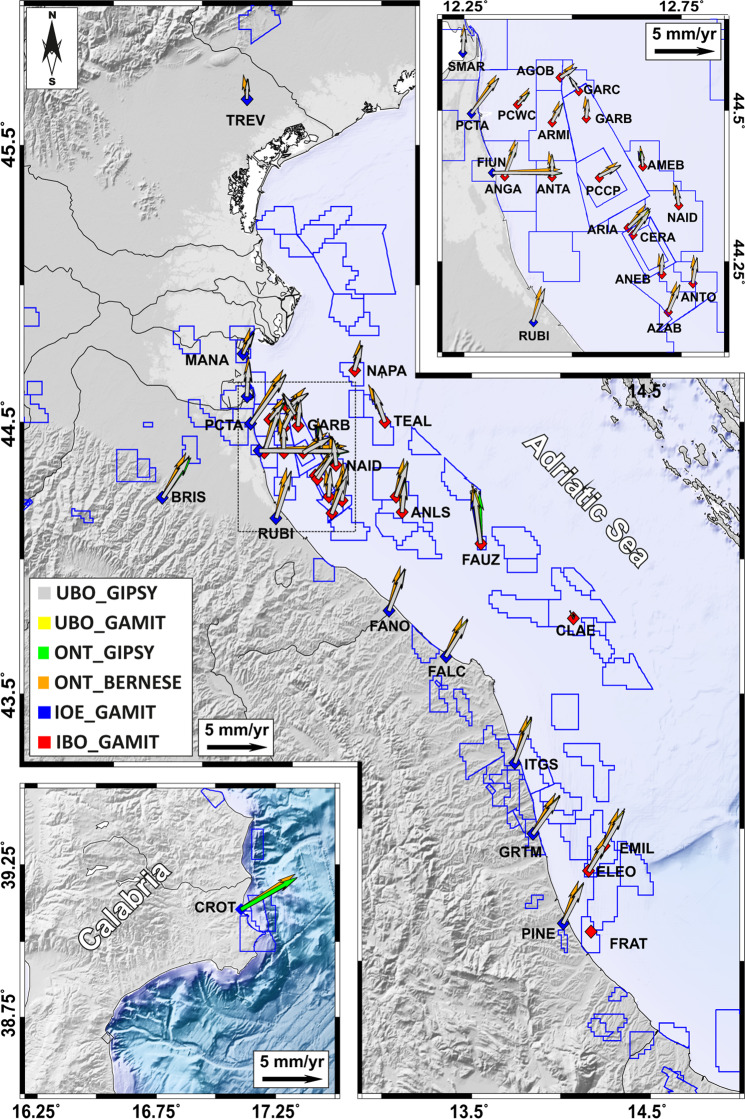
Horizontal velocity field comparisons. Velocities are referred to a Eurasian^35^ reference frame. Lower inset: zoom on Calabria region; upper inset: zoom on the Northern Adriatic region.

As a second step, we computed for each solution the velocity field with respect to a Eurasian^35^ reference frame; achieved results are reported in Fig. 6. Because of its simple definition, such a rotation affects only the horizontal velocities; therefore, the vertical ones remained constrained in their previous reference frame. Considering the horizontal velocity (Fig. 6), all the solutions show a general agreement in terms of azimuthal pattern and vector magnitudes, however the ONT_BERNESE solution show a systematic counterclockwise rotation with respect to the other ones, probably related to the Helmert constraints imposed for the reference frame transformation (Table 2). The ONT_GIPSY solution, although referred to IGS14, is highly coherent with the other solutions. Most of the stations installed along the onshore coastal area show a prevailing NNW-oriented velocity pattern which is highly coherent with the regional deformation field^14,27,36^. Stations installed along the Adriatic offshore show a complex deformation field characterized by large variations both in the azimuthal pattern and the vector magnitudes (Fig. 6).

**Fig. 7:**
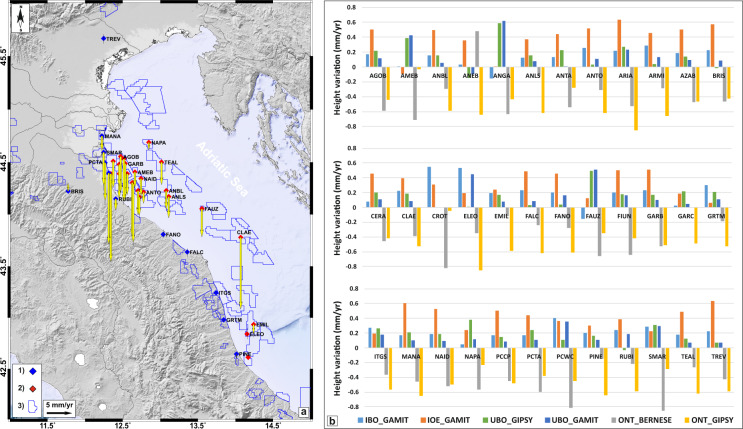
(**a**) mean vertical velocity field 5; (**b**) comparison of the residual vertical velocity values for all the analyzed solutions.

The vertical velocities are reported in Fig. 7 as averaged values (panel a) and as differences with respect to the average values (panel b). Figure 7a shows that most of the stations installed along the onshore coastal area are characterized by subsidence with rates up to 2 mm/yr in agreement with recent studies^8^, while all the stations installed on the offshore platforms exhibit a general subsidence with rates up to ~17 mm/yr (see also Table 6). The site-by-site comparisons reported in Fig. 7b confirm the features previously recognized: the ONT_GIPSY and ONT_BERNESE solutions are generally smaller than the mean values, while the other solutions are larger than the mean values. These differences seem related to the adopted reference (the ONT_GIPSY solution is referred to IGS14) and/or to the constraints imposed for the reference frame transformation (the ONT_BERNESE solution adopts a Helmert transformation based only on 4-parameters).

### Final considerations

As mentioned above, computed position time series were analyzed by means of the TSAnalyzer^30,31^ open-source software in order to estimate the linear and seasonal components as well as the offsets values.

We detected some offsets corresponding to equipment changes and to co-seismic deformation. Moreover, some detected offsets, classified as “unknown” in Table 3, are characterized by motion probably related to service operations on platform. All detected offsets have been identified in all solutions and are characterized by very similar values (see related log files on the online repository).

Examined position time series contain significant seasonal signals: annual amplitudes range between 0.5–5.3 mm horizontally and 3–8 mm vertically while semi-annual amplitudes are usually < 0.5 mm horizontally and ~1 mm vertically. Higher annual amplitude values were observed only at sites installed on the offshore platforms, with maximum amplitudes on January, September and August for North, East and Up components, respectively. The horizontal oscillations occur with a prevailing NNW-SSE attitude while the vertical oscillation are associated with uplift during summer and subsidence during winter. Conversely, sites installed along the coastal area are characterized by maximum amplitudes largely scattered and no clear oscillation patterns can be recognized. All these observations suggest that offshore stations would be affected by variations in hydrostatic pressure and buoyancy on tubular members of the platforms caused by changes in tide levels. Moreover, since the Adriatic Sea is an almost land-locked basin where atmospheric conditions and vary considerably with the seasons^37,38^, environmental loads caused by wind (e.g. periodic seiche-like effects), current flows and waves would substantially contribute to the observed seasonal oscillations. Due to the main metallic-fabric of platform, seasonal thermal expansion-contraction cycles of the tiny structures could also contribute to the seasonal oscillations.

A large number of stations is characterized also by position time series with non-linear behavior at one or more components, as quantified by the computed *I*_*NL*_ index (Table 5). Stations with *I*_*NL*_ values larger than 1 are generally characterized also by high subsidence rates (Table 6 and Fig. 7), clearly suggesting that, the tectonic deformation is superseded by the local sources ones (e.g. reservoir depletion, sediment compaction, etc.). Moreover, these local sources affected also the horizontal deformation rates leading to a complex pattern, strictly depending by the relative position of the station (i.e. the platform) with respect to the sources of deformation. Similar considerations can be done for the stations with 0.5 ≤ *I*_*NL*_ ≤ 1, where however, the effects of local sources have had a minor influence on station motion. Conversely, stations with *I*_*NL*_ < 0.5 show a velocity pattern which is highly coherent with the regional deformation field of the investigated area.

Based on the analysis and the comparisons performed in this study, the different time series solutions are highly consistent between them despite the use of different software, models, strategy processing and frame realizations. The analyzed dataset represents an invaluable dataset since it allows to improve the current GNSS stations density along the Adriatic coastal belt. In addition, this dataset allowed to discover a complex interplay between regional and local sources of deformation, whose relationships would be addressed in future analysis. Indeed, because of their uniqueness, these data could improve and promote further studies in offshore exploiting contexts including: (i) measurement and modelling of induced subsidence patterns and their spatial and temporal correlation with production data, (ii) estimation of natural and anthropic contributions to the overall ground subsidence, (iii) improvement on the long-term regional crustal deformation on offshore areas, (iv) coast-line dynamic and impact on human activities and natural ecosystems (e.g. coast line setback).

## Supplementary information

Supplementary Information
