# Supplementary material for: Geopositioning time series from offshore platforms in the Adriatic Sea
Source: Sci Data. 2020 Nov 4;7:373. doi: 10.1038/s41597-020-00705-w (PMC7643151; doi:10.1038/s41597-020-00705-w)
Supplement: Supplementary file 1 — Supplementary Information [file 41597_2020_705_MOESM1_ESM.docx]

**Raw RINEX Data Access**

Interested scientific users (Universities, Research Institutes, Governmental Scientific Agencies, etc.) can obtain a free access to data by contacting Eni S.p.A. company. Private companies can also access to the data, however, to protect own commercial interests, Eni S.p.A. will consider applications each time. Applicants have to contact Eni S.p.A. at the contact point (Dr. Marco Mileti, [marco.mileti@eni.com](mailto:marco.mileti@eni.com)).

Applicants (Principal Investigator) must submit a research project clearly describing:

- Aim of the project;
- The analyses which will be performed on the raw data;
- Additional partners involved in project.

When all of the required documentation has been received by ENI S.p.A., the submission will be assigned to a contracts officer for review and negotiation. Once the terms have been finalized by the contracts officer, a “Data User Agreement” (DUA) will be circulated for signature (see the copy reported in the following). Once the DUA has been fully executed (signed by all parties), the access to raw data will be provided to the Principal Investigator.

A copy of the DUA is reported in the following. Additional statements/points could be inserted on the confidential agreement based on i) the nature/scope of the applicant requests and ii) changes of the data policy of Eni S.p.A. company.

**DATA USE AGREEMENT**

This Data Use Agreement (DUA), effective as of __________, 20__, is entered into by and between ENI S.p.A. and _____________________ (Data User). The purpose of this DUA is to provide a free access to a dataset for use in the following titled research project: ___________________________________ (Project Name) under the direct supervision of ________________________________ (Principal Investigator).

In consideration of the mutual promises and covenants, herein, and for other good and valuable consideration, the receipt and sufficiency of which is hereby acknowledged, ENI S.p.A. and Data User agree as follows:

1. The dataset consists in (brief description of the requested dataset): ___________________________ ____________________________________________________________________________________________________________________________________________________________________________________________________________________________________________________________________________________________________________________________________
2. The dataset is made available on a non-exclusive basis and is confidential; Eni S.p.A. will retain ownership of the dataset and may request its return at any time with written communication sent to the Principal investigation. The dataset will be returned, and any related written or electronic copies destroyed, within 30 days of receiving such notice.
3. Data User may terminate this agreement at any time by notifying Eni S.p.A. and returning or destroying the dataset.
4. The dataset can be used only in the framework of the following research project (project title and brief description): __________________________________________________________________

___________________________________________________________________________________________________________________________________________________________________________________________________________________________________________________

1. Data User agrees to keep the dataset strictly confidential; the dataset cannot be published or shared to third-parties. Achieved results can be shared/published/disclosed all after Eni S.p.A. written consent. Data User (or his legal representative) will be responsible for any illegal use.
2. External collaborators involved in the framework of the Project can access to the dataset under the same conditions and restrictions of Data User. Authorized external collaborators are (provide: name, surname, Institution, role in the framework of the project):
3. __________________________________________________________________________
4. __________________________________________________________________________
5. __________________________________________________________________________
6. …….
7. Data User agrees to indemnify and hold harmless ENI S.p.A. from and against all claims, demands, liabilities, judgments, or causes of action of any nature for any relief, elements of recovery or damages recognized by Italian law (including, without limitation, attorney’s fees, defense costs, and equitable relief), for any damage or loss arising out of, resulting from, or attributable to any acts or omissions or other as a result of the violation of this agreement.
8. Eni S.p.A. will have no liability for any use of the dataset by the Data User and the authorized external collaborators.
9. Eni does not provide any guarantee, expressed or implied, regarding the quality, accuracy and completeness of the dataset; Data User (or his legal representative) acknowledges the implicit risk of errors in the acquisition, processing and interpretation of requested dataset.
10. This data user agreement shall be governed by and interpreted in accordance with the Italian laws.

In WITNESS WHEREOF, each of the undersigned has caused this Agreement to be duly executed in its name and on its behalf.

| **ENI S.p.A.**  By: ____________________________  (Authorized Signature)  Name: _________________________  (Type or Print)  Title:___________________________  Date:___________________________ | **Data User**  By: ____________________________  (Authorized Signature)  Name: _________________________  (Type or Print)  Title:___________________________  Date:___________________________ |
| --- | --- |
